# Temporal and spatial changes in rhizosphere bacterial diversity of mountain *Rhododendron mucronulatum*

**DOI:** 10.3389/fmicb.2023.1201274

**Published:** 2023-06-21

**Authors:** Sirui Wang, Tiantian Zhou, Hewen Zhao, Kezhong Zhang, Jinteng Cui

**Affiliations:** ^1^College of Landscape Architecture, Beijing University of Agriculture, Beijing, China; ^2^Ancient Tree Health and Culture Engineering Technology Research Center, Beijing University of Agriculture, Beijing, China

**Keywords:** *Rhododendron mucronulatum*, rhizosphere soil, rhizosphere bacteria, bacterial diversity, *Rhizomicrobium*

## Abstract

To better conserve the ecology of the wild *Rhododendron mucronulatum* range, we studied the rhizosphere microenvironment of *R. mucronulatum* in Beijing’s Yunmeng Mountain National Forest Park. *R. mucronulatum* rhizosphere soil physicochemical properties and enzyme activities changed significantly with temporal and elevational gradients. The correlations between soil water content (SWC), electrical conductivity (EC), organic matter content (OM), total nitrogen content (TN), catalase activity (CAT), sucrose-converting enzyme activity (INV), and urease activity (URE) were significant and positive in the flowering and deciduous periods. The alpha diversity of the rhizosphere bacterial community was significantly higher in the flowering period than in the deciduous period, and the effect of elevation was insignificant. The diversity of the *R. mucronulatum* rhizosphere bacterial community changed significantly with the change in the growing period. A network analysis of the correlations revealed stronger linkages between the rhizosphere bacterial communities in the deciduous period than in the flowering period. *Rhizomicrobium* was the dominant genus in both periods, but its relative abundance decreased in the deciduous period. Changes in the relative abundance of *Rhizomicrobium* may be the main factor influencing the changes in the *R. mucronulatum* rhizosphere bacterial community. Moreover, the *R. mucronulatum* rhizosphere bacterial community and soil characteristics were significantly correlated. Additionally, the influence of soil physicochemical properties on the rhizosphere bacterial community was larger than that of enzyme activity on the bacterial community. We mainly analyzed the change patterns in the rhizosphere soil properties and rhizosphere bacterial diversity of *R. mucronulatum* during temporal and spatial variation, laying the foundation for further understanding of the ecology of wild *R. mucronulatum*.

## 1. Introduction

*Rhododendron mucronulatum*, a deciduous shrub of the Ericaceae, has excellent cold hardiness and poor heat tolerance (Liu et al., 2022). *R. mucronulatum* is an important flowering plant in early spring in northern China. However, it is only widely distributed in the Yan Mountains in the Beijing area, rarely appearing in other areas. Soil is a complex and dynamic ecosystem containing many microorganisms (Bryant et al., 2008; Liu et al., 2021). The distribution and activities of microorganisms in soil reflect not only the influence of soil factors on the ecological distribution and biochemical properties of microorganisms but also the regulatory role of microorganisms on plant growth and development, material cycling, and energy transformation (Bartelt-Ryser et al., 2005). The study of soil microbial diversity can reveal the ecological function of microorganisms and the change pattern of the soil environment, which is closely related to soil ecological stability (Hicks et al., 2021). Rhizosphere microorganisms inhabit the surface of plant roots and are one of the most complex microbial communities on Earth (Shi et al., 2021). Understanding changes in the rhizosphere microbial community is important for the evolution of the plant community. Rhizosphere microorganisms are interdependent with plants, and rhizosphere bacteria produce volatile organic compounds that mediate bacteria–plant interactions (Bailly and Weisskopf, 2012; Zuo et al., 2022a). Plants produce a range of carbon metabolites as microbial food and energy sources (Olanrewaju et al., 2019). Rhizosphere microorganisms can promote plant growth by improving soil nutrients, increasing the supply of plant mineral nutrients, and promoting the synthesis of plant secondary metabolites (Fan et al., 2020; Shi et al., 2021). For example, rhizosphere bacteria can accelerate the dissolution of insoluble phosphates in soil, convert nitrogen into ionic ammonia, and promote plant absorption (Majeed et al., 2018; Barney, 2020).

Through comparative analysis of microbial communities, many scholars have found that microorganisms can play an important role in promoting plant growth. For example, Islam et al. (2023) found that *Wandonia haliotis*, *Singulisphaera rosea*, and *Zhizhongheella caldifontis* could be indicator species for *Trillium govanianum* growth promoters. Xu et al. (2022) found that *Haliangium* and *Candidatus Koribacter* could protect *Scutellaria tsinyunensis* by controlling combinations of multiple covariates. Further, Zamioudis and Pieterse (2012) posited that beneficial rhizosphere microorganisms can promote plant growth by degrading soil pollutants or inhibiting plant diseases and pests. However, there is no study systematically addressing the profile of microorganisms inhabiting the rhizosphere of Rhododendron mucronulatum yet.

The diversity of rhizosphere bacterial communities depends on soil characteristics and host plant types (Yuan et al., 2014). The non-biological characteristics of soil, including soil pH, electrical conductivity (EC), organic matter, nitrogen, phosphorus, and potassium, can also significantly affect plants (Hu et al., 2019). Therefore, we investigated the rhizosphere bacterial diversity of *R. mucronulatum* in Yunmeng Mountain National Forest Park and analyzed the relationship between the temporal and spatial variation and the rhizosphere soil characteristics and rhizosphere bacterial diversity of *R. mucronulatum*. Our findings deepen the current understanding of the rhizosphere microenvironmental variation patterns of *R. mucronulatum*, which is ecologically important for conserving the distribution of wild *R. mucronulatum* in Yunmeng Mountain National Forest Park.

## 2. Materials and methods

### 2.1. Soil collection

Yunmeng Mountain is at the southern foot of the Yan Mountains, a rainy zone in Beijing. The annual precipitation is approximately 700 mm, and erosion due to flowing water is strong. The total area is 2,208 ha. The study site is located in Yunmeng Mountain National Forest Park (40°26′–40°38′N, 116°30′–116°50′E), with the highest peak elevation up to 1,414 m. According to the distribution of wild *R. mucronulatum*, the elevation range at 600–1,414 m was divided into three experimental areas: high elevation (1,214–1,386 m), middle elevation (933–961 m), and low elevation (652–647 m). Nine well-grown *R. mucronulatum* plants were selected from wild *R. mucronulatum* populations in different experimental areas in spring (mid-April) and autumn (late October), and 20 cm of the root was dug out. The soil attached to the root was shaken off to derive soil samples for the nine replicates. These samples were collected and placed in sterile sealed bags. Every sample was divided into three parts. The first part, was used to analyze rhizosphere bacterial diversity. The second part, was used to determine enzyme activity in soil. The third part was used to determine soil physicochemical properties. All three parts were stored in a −80°C refrigerator.

### 2.2. Determination of the integrated physicochemical and enzymatic activities of *R. mucronulatum* rhizosphere soil

Soil physicochemical and enzymatic activity indicators included soil water content (SWC), pH, EC, organic matter content (OM), and total nitrogen content (TN), as well as catalase activity (CAT), sucrose-converting enzyme activity (INV), and urease activity (URE). SWC was calculated after drying at 105°C overnight (Jiao et al., 2023). The pH was measured with a pH meter (Mettler Toledo) in a 1:2.5 soil:water suspension (Zhang et al., 2013). The EC was measured with a conductivity meter (Mettler Toledo) in a 1:5 soil:water suspension(Cruz-Paredes et al., 2021). The OM was determined using the K_2_Cr_2_O_7_ oxidation method (Wang et al., 2022a). The TN was determined using the Kjeldahl method (Guo et al., 2020). Further, the CAT was determined through titration with KMnO_4_ (Wang et al., 2022a). The INV was determined using a 3,5-dinitrosalicylic acid colorimetric assay (Wang et al., 2022a). Finally, the URE was determined via indophenol blue colorimetric method (Yang et al., 2021).

### 2.3. DNA extraction, polymerase chain reaction amplification, and illumina sequencing

Total rhizosphere soil DNA was extracted using a Soil Genomic DNA Kit (CoWin, JiangSu, China) and stored at −20°C for subsequent sequencing. The V4 region of the 16S rRNA gene (515F,5′-CAGCMGCCGCGGGTAA-3′; 806R:5′-GACTACHVGGGTWTCTAAT-3′) was amplified via polymerase chain reaction (PCR) (Youssef et al., 2009; Caporaso et al., 2011). Thermal cycling was performed under the following conditions: 98°C for 1 min, 30 cycles of 98°C for 10 s, 50°C for 30 s, 72°C for 30 s, and a final extension at 72°C for 5 min. Sequencing libraries were generated using a TruSeq DNA PCR-Free Sample Preparation Kit (Illumina, United States), and index codes were added. The library quality was assessed on the Qubit@ 2.0 Fluorometer (Thermo Scientific) and Agilent Bioanalyzer 2,100 system. Last, the library was sequenced on an Illumina NovaSeq platform, and 250 bp paired-end reads were generated.

### 2.4. Bioinformatics analysis

Paired-end reads were merged using FLASH (v1.2.7; Magoc and Salzberg, 2011). Quality filtering was performed to produce high-quality clean reads from raw reads according to the QIIME (v1.7.0) quality control process (Caporaso et al., 2010). Chimeric sequences were selected from the Gold database through comparison (version microbiomeutil-r20110519, http://drive5.com/uchime/uchime_download.html), which were then removed using uchime. Sequences were assigned to operational classification units (OTUs) using UPARSE (V7.0.1001) at a 97% homology threshold. Species annotation analysis was carried out using the Mothur method with SILVA’s SSUrRNA database to obtain taxonomic information and to count the community composition of each sample separately at each taxonomic level (Wang et al., 2007; Quast et al., 2013). Based on the annotation results, a relative abundance map at the phylum level was plotted using perl-SVG. The alpha diversity indicated by the Shannon and Chao1 indices was calculated with QIIME (v1.7.0). Furthermore, QIIME (v1.7.0) was used to calculate the UniFrac distance. We created the PCoA plot using R (Version 2.15.3) software. An analysis of a similarity randomization test (ANOSIM) was performed to determine whether the bacterial composition differed among different treatment groups. Then, a linear discriminant analysis of the effect size (LEfSe) was performed at every rank of classification to estimate differentially represented bacteria using a logarithmic linear discriminant analysis threshold score of 4.0 (Segata et al., 2011). The genera (top 50) were selected for Spearman’s correlation index calculation (*p* < 0.01), and the genera correlation coefficient matrix was subject to the following filtering conditions: (1) remove connections with correlation coefficients of <0.6, (2) filter node self-connections, and (3) remove connections with node abundance of less than 0.005%. Spearman correlation coefficient values of strains (top 35) and environmental factors were calculated and tested for significance using the corr.test function in R (Version 2.15.3).

### 2.5. Statistical analysis

Violin plots of EC, pH, SWC, OM, TN, CAT, INV, and URE data and box plots of the Shannon index and Chao1 index were generated using GraphPad Prism 8. Statistical analysis was performed using SAS (8.01, SAS Institute, Inc., Cary, NC, United States) software. A one-way analysis of variance was used to test for differences in rhizosphere soil physicochemical properties and enzymatic activities between different elevations and growth periods, and Duncan’s multiple polar difference test (*p* < 0.05) was performed when significant differences were detected between the means. Pearson correlation analysis was used to statistically analyze the correlation between the physicochemical properties and enzymatic activities of rhizosphere soil. Chao1 and Shannon indices were used to detect between-group differences in treatment groups using a *t-*test.

## 3. Results

### 3.1. Analysis of the integrated physicochemical properties and enzymatic activities of *R. mucronulatum* rhizosphere soil

**Figure 1:**
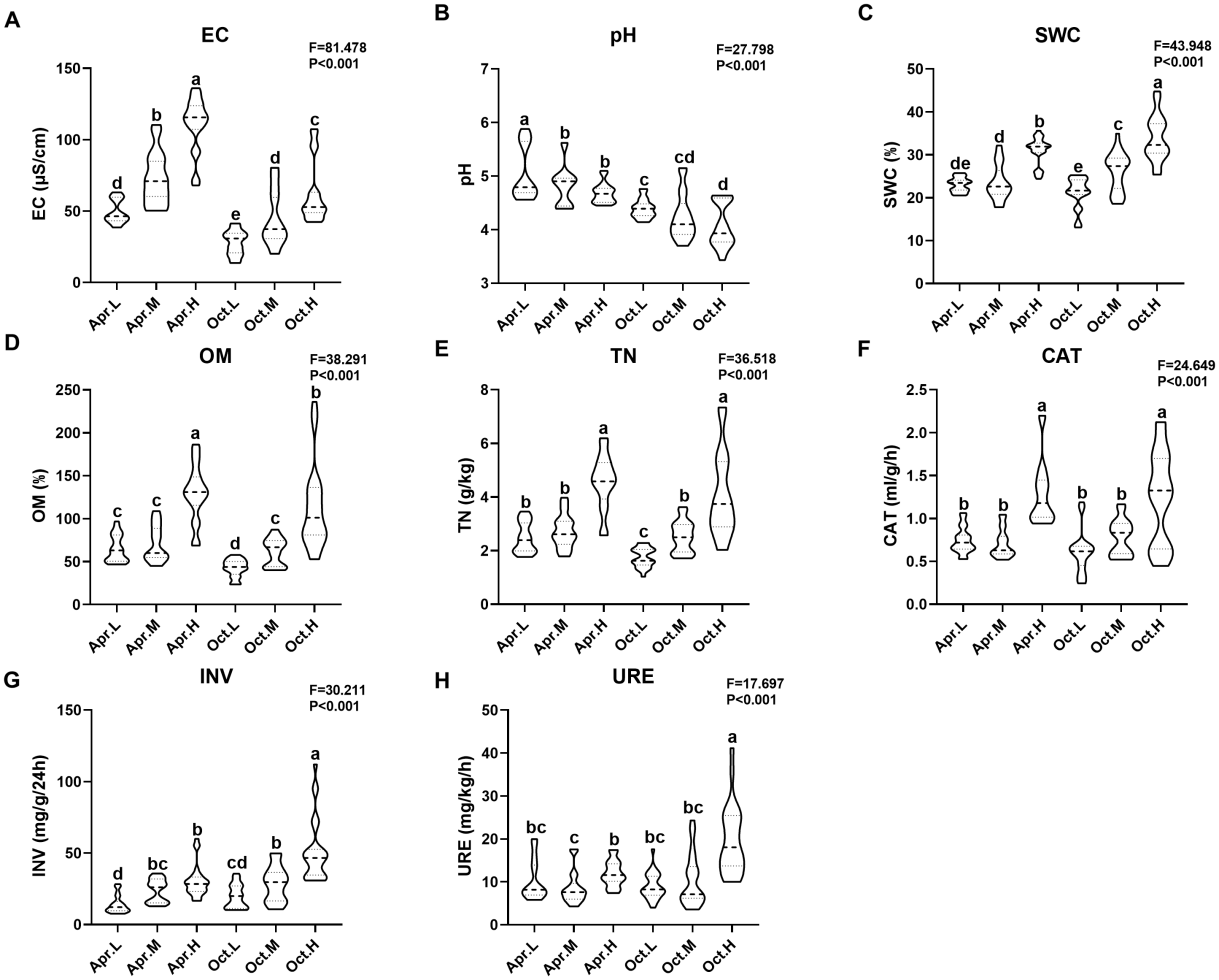
Analysis of integrated physicochemical properties and enzymatic activities of *R. mucronulatum* rhizosphere soil. The violin frame shows the kernel density of the data distribution. Apr.L, flowering period low elevation; Apr.M, flowering period medium elevation; Apr.H, flowering period high elevation; Oct.L, deciduous period low elevation; Oct.M, deciduous period medium elevation; Oct.H, deciduous period high elevation; EC, electrical conductivity; pH, soil pH; SWC, soil water content; OM, organic matter; TN, total nitrogen; CAT, soil catalase; INV, soil sucrose converting enzyme; URE, soil urease. **(A–H)** represent EC, pH, SWC, OM, TN, CAT, INV, and URE. *F* and *P* represent the ANOVA test results for the entire elevation. Duncan’s multiple extreme difference test was used to compare the significant differences between the two samples (*p* < 0.05).

We measured physiological and biochemical indicators, including EC, pH, SWC, OM, TN, CAT, INV, and URE, and organized the data into a violin plot (Figure 1). *R. mucronulatum* rhizosphere soil physicochemical properties (EC, pH, SWC, OM, and TN) and enzyme activities (CAT, INV, and URE) changed significantly (*p* < 0.001) over temporal and elevational gradients. Among them, EC, SWC, OM, TN, CAT, and INV increased significantly with increasing elevation (Figures 1A,C–G). Soil pH decreased significantly with increasing elevation (Figure 1B). Soil SWC, OM, TN, and CAT did not differ significantly at low and medium elevations, but the values at high elevations were significantly higher than those at low and medium elevations. Soil URE in deciduous period was significantly higher at high elevations than at low and middle elevations (Figure 1H). Soil EC increased significantly with increasing elevation in flowering period. The rhizosphere soil EC and pH were significantly higher in the flowering period than in the deciduous period at different periods and at the same elevation, and the difference in CAT activity between the flowering and deciduous periods was not significant.

Correlation analysis was performed using physicochemical indicators and enzymatic activity of *R. mucronulatum* rhizosphere soil. As shown in Table 1, SWC, EC, OM, TN, CAT, INV, and URE significantly positively correlated at both the flowering and deciduous periods, except for INV and URE, which were only significantly positively correlated at the deciduous period. In the deciduous period, pH significantly negatively correlated with SWC. In the flowering period, pH significantly negatively correlated with INV, significantly positively correlated with URE, and insignificantly correlated with other physiological and biochemical indicators. In this experiment, there was a close correlation between every physiological and biochemical index, with the exception of pH.

**Table 1:** Correlation analysis between integrated physicochemical properties and enzymatic activity of *R. mucronulatum* rhizosphere soil.

	SWC	pH	EC	OM	TN	CAT	INV
pH	−0.206						
	−0.297**						
EC	0.760**	0.027					
	0.705**	−0.047					
OM	0.817**	−0.035	0.887**				
	0.721**	−0.069	0.906**				
TN	0.714**	0.068	0.744**	0.819**			
	0.681**	−0.030	0.871**	0.946**			
CAT	0.684**	0.150	0.859**	0.854**	0.744**		
	0.422**	−0.056	0.610**	0.641**	0.611**		
INV	0.756**	−0.284*	0.570**	0.686**	0.583**	0.431**	
	0.579**	−0.060	0.848**	0.883**	0.886**	0.581**	
URE	0.331**	0.534**	0.558**	0.572**	0.530**	0.737**	0.216
	0.458**	0.209	0.738**	0.679**	0.705**	0.572**	0.711**

*p < 0.05; **p < 0.01. The upper layer is the correlation of flowering period and the lower layer is the correlation of deciduous period.

### 3.2. Composition and classification of *R. mucronulatum* rhizosphere bacteria

**Figure 2:**
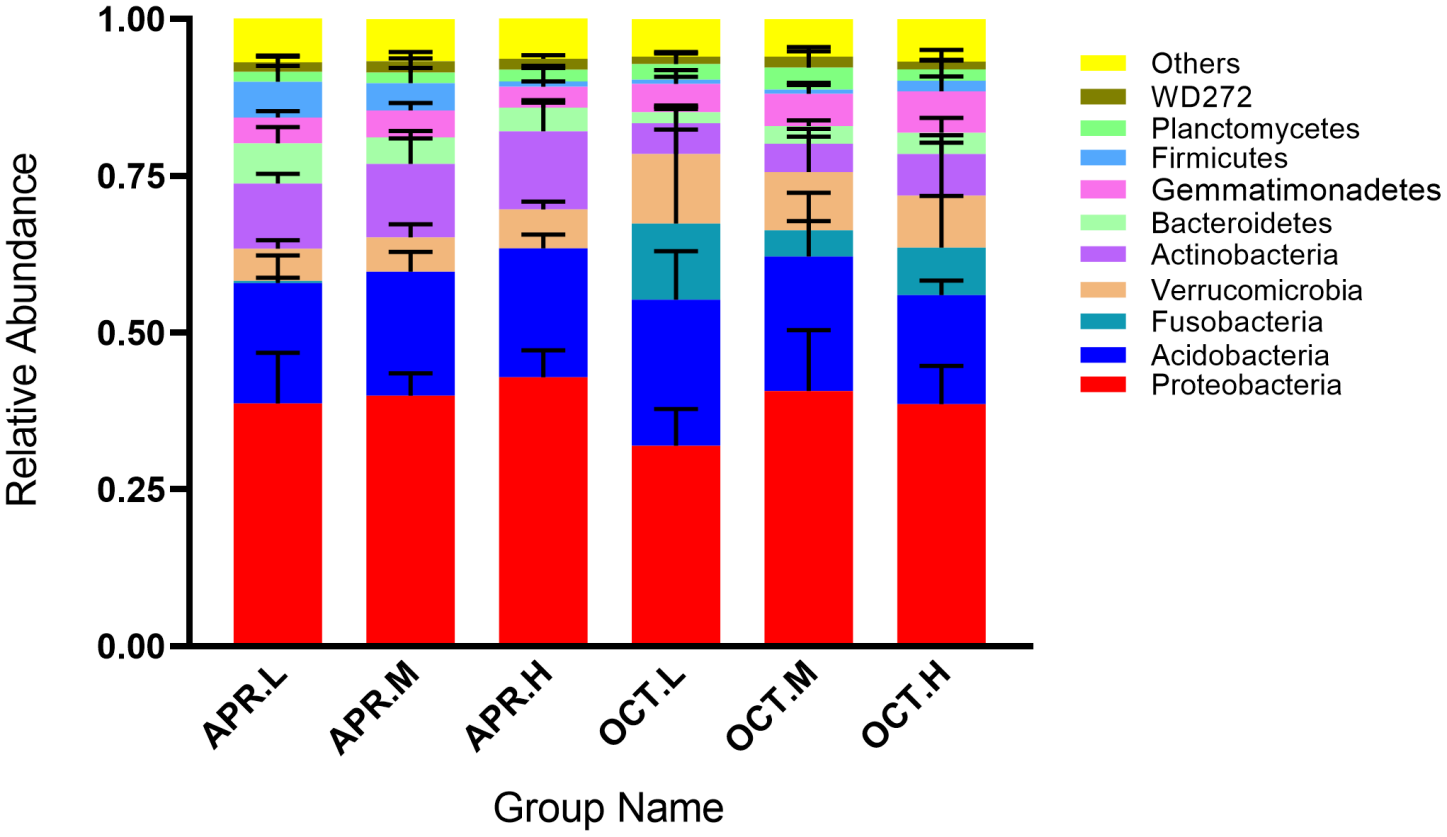
Relative abundance of *R. mucronulatum* rhizosphere bacterial at phylum levels. Only the top 10 bacteria phylum were presented. Apr.L, flowering period low elevation; Apr.M, flowering period medium elevation; Apr.H, flowering period high elevation; Oct.L, deciduous period low elevation; Oct.M, deciduous period medium elevation; Oct.H, deciduous period high elevation.

As shown in Figure 2, the top 10 average abundant phyla are Proteobacteria (38.80%), Acidobacteria (20.27%), Actinobacteria (8.44%), Verrucomicrobia (7.59%), Gemmatimonadetes (4.68%), Fusobacteria (4.03%), Bacteroidetes (3.72%), Firmicutes (2.31%), Planctomycetes (2.15%), and WD272 (1.56%). Where Proteobacteria, Acidobacteria, Actinobacteria, and Verrucomicrobia were the dominant phyla (relative abundance >5%) among *R. mucronulatum* rhizosphere bacteria. The relative abundance of Proteobacteria in the rhizosphere soil flowering period reached a maximum at high elevations (42.89%), and the relative abundance of Proteobacteria in the rhizosphere soil in deciduous period reached a maximum at middle elevations (40.70%). The relative abundance of Proteobacteria at flowering period was higher than at deciduous period. The relative abundance of Acidobacteria in rhizosphere soil in flowering period increased with elevation, while the relative abundance of Acidobacteria in rhizosphere soil in deciduous period decreased with elevation. The relative abundance of Acidobacteria in the rhizosphere soil in the deciduous period was higher than that in the flowering period. The relative abundance of Actinobacteria in the rhizosphere soil in the flowering stage was higher than that in the deciduous period and reached a maximum at high elevations. The relative abundance of Verrucomicrobia in the rhizosphere soil in the deciduous period was higher than that in the flowering period and reached a maximum at low elevations. The relative abundance of Fusobacteria in the rhizosphere soil reached the top 10 only in the deciduous period and was extremely low in the flowering period. The effect of growth period on the relative abundance of rhizosphere bacteria was higher than the effect caused by the change in elevation.

**Figure 3:**
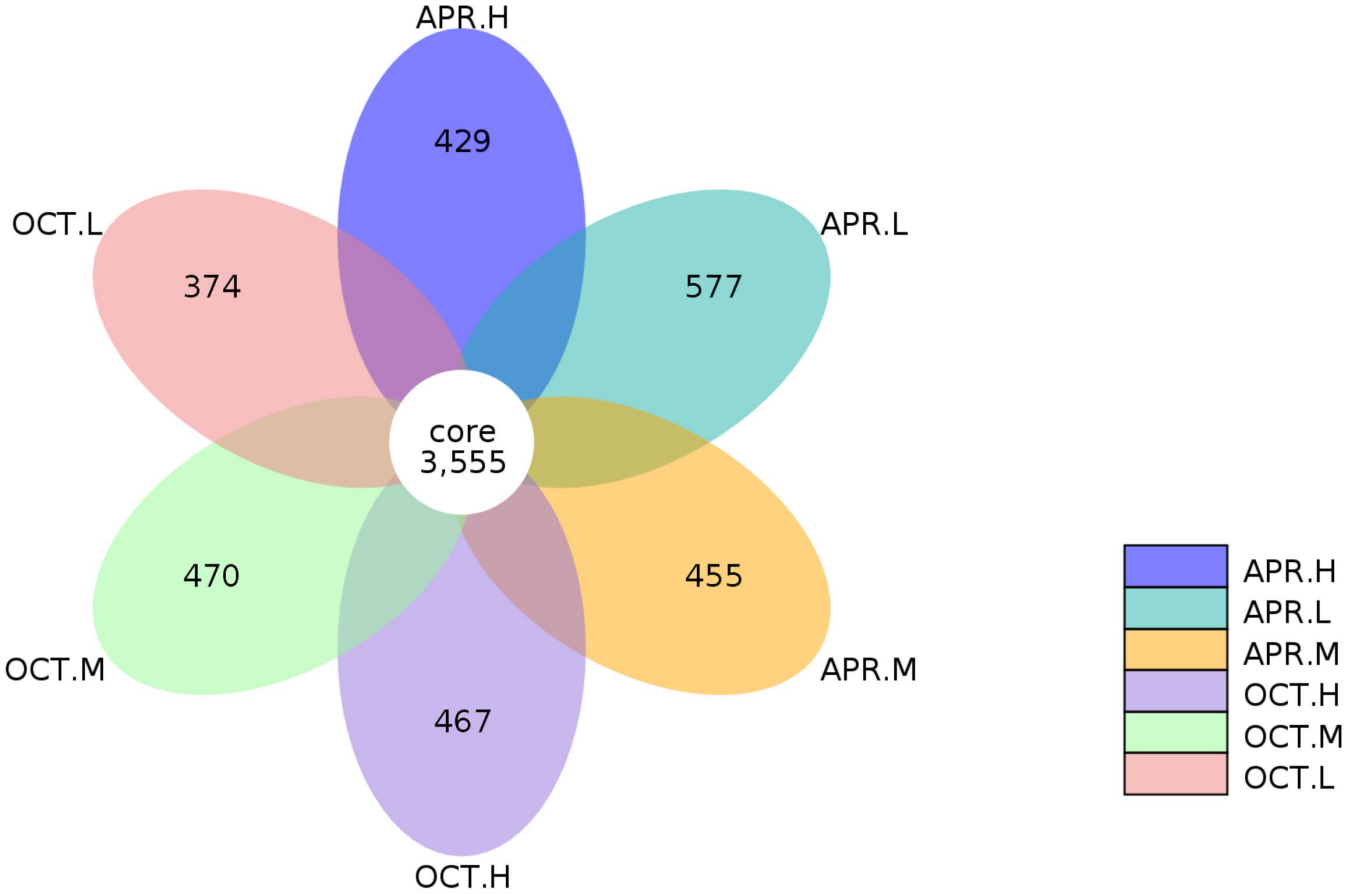
Petal diagram of *R. mucronulatum* rhizosphere bacterial OTUs. Petal diagram showing the number of bacterial operational taxonomic units (OTUs) at different growth periods and elevations. Apr.L, flowering period low elevation; Apr.M, flowering period medium elevation; Apr.H, flowering period high elevation; Oct.L, deciduous period low elevation; Oct.M, deciduous period medium elevation; Oct.H, deciduous period high elevation.

We analyzed the OTUs common to and unique to the samples from various treatment groups. As shown in Figure 3, the OTUs of all of the sample groups were clustered, and a total of 6,327 OTUs were obtained. The number of OTUs endemic to *R. mucronulatum* floral rhizosphere bacteria was 577, 455, and 429, accounting for 13.96%, 11.34%, and 10.77% of the total OTUs contained at low, middle, and high elevations, respectively, in the floral period. The number of OTUs endemic to rhizosphere bacteria at the deciduous period was 374, 470, and 467 from low to high elevations, accounting for 9.52%, 11.68%, and 11.61% of the total OTUs contained at low, middle, and high elevations, respectively, in the deciduous period. At lower elevations, the number of OTUs specific to rhizosphere bacteria in flowering period was significantly higher than at other elevations, and the number of OTUs specific to rhizosphere bacteria in deciduous period was significantly lower than at other elevations. The species composition of rhizosphere bacteria differed temporally and spatially.

### 3.3. Diversity and richness of *R. mucronulatum* rhizosphere bacterial communities

**Figure 4:**
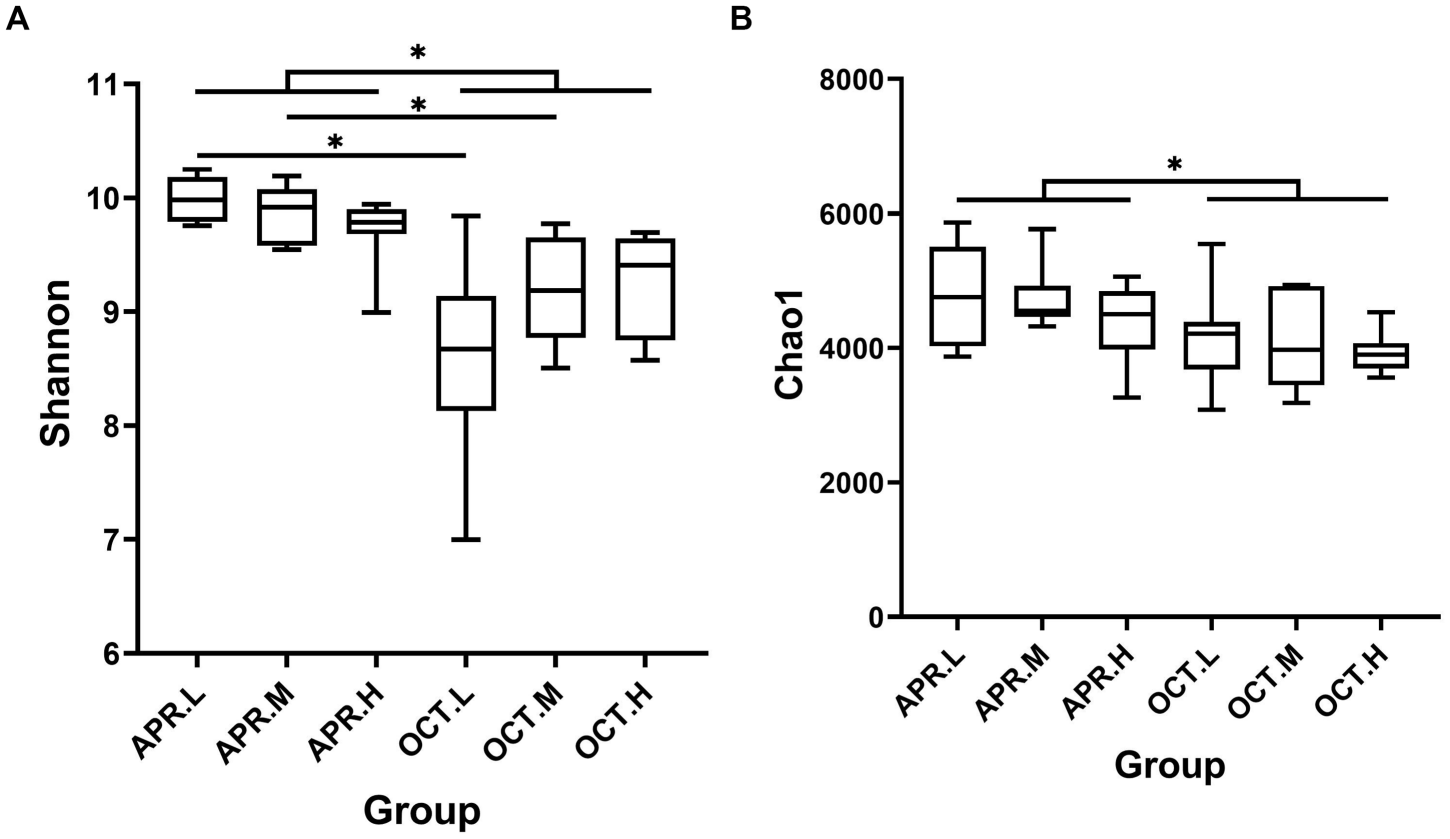
Analysis of inter-group differences in Shannon **(A)** and Chao1 **(B)** indices (*n* = 9 per treatment) of *R. mucronulatum* rhizosphere bacteria. The line in the middle of the box in the box plot represents the median, and the horizontal line above the box indicates the significance level of the difference between the two sets of data (*p < 0.01). Apr.L, flowering period low elevation; Apr.M, flowering period medium elevation; Apr.H, flowering period high elevation; Oct.L, deciduous period low elevation; Oct.M, deciduous period medium elevation; Oct.H, deciduous period high elevation.

We analyzed bacterial community diversity and richness using the alpha diversity indices Shannon and Chao1 (Figure 4). In the Shannon index box plot (Figure 4A), the diversity of rhizosphere bacterial communities was significantly higher during the flowering period than during the deciduous period. There were significant differences in Shannon indices in different growth periods at low and medium elevations. At the same growth period, there was no significant difference in rhizosphere bacterial community diversity among different elevation. In the Chao1 index box plot (Figure 4B), the richness of rhizosphere bacterial communities was significantly higher during the flowering period than during the deciduous period. At the same growth period, there was no significant difference in rhizosphere bacterial community richness among different elevation. The growth period had a larger effect on the richness of rhizosphere bacterial communities than elevation. The diversity and community richness of rhizosphere bacteria were higher in the flowering period than in the deciduous period, possibly owing to the high metabolism of plants in the flowering period and more rhizosphere secretion, which provided nutrients for the growth and reproduction of rhizosphere bacteria and increased the species and number of bacteria. During the flowering period, the bacterial activity in the soil increased with increasing ground temperature, and the rhizosphere bacterial activity was active. As *R. mucronulatum* entered the deciduous period, the environmental temperature decreased, the Shannon index decreased, and bacterial diversity decreased, which may be related to the decrease in the plant metabolism rate and changes in root exudation produced.

### 3.4. Differential analysis of *R. mucronulatum* rhizosphere bacteria

**Figure 5:**
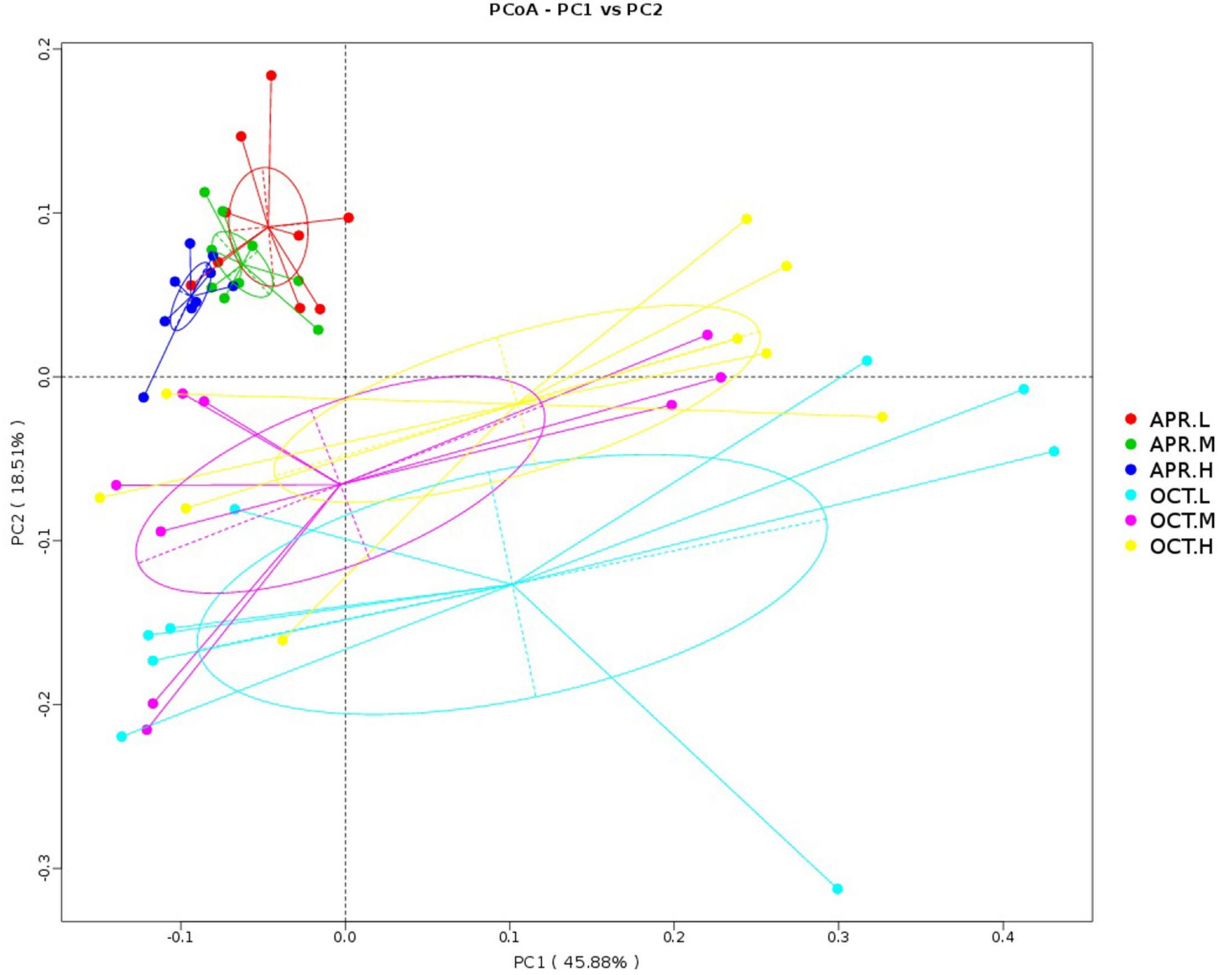
PCoA analysis of the rhizosphere bacterial community of *R. mucronulatum*. PCoA analysis based on principal coordinates analysis of weighted UniFrac distances (*n* = 9 per treatment), showing changes in the rhizosphere bacterial community of *R. mucronulatum* occurring at different growth periods and elevations. The colors of the dots and lines represent different growth periods and elevations. Apr.L, flowering period low elevation; Apr.M, flowering period medium elevation; Apr.H, flowering period high elevation; Oct.L, deciduous period low elevation; Oct.M, deciduous period medium elevation; Oct.H, deciduous period high elevation.

We compared the composition of rhizosphere bacterial communities using PCoA. As shown in Figure 5, the principal component axes of *R. mucronulatum* rhizosphere bacteria of various temporal and spatial samples were 45.88% for PC1 and 18.51% for PC2. Significant differences existed in the rhizosphere bacterial community during the flowering and deciduous periods. At different growth periods in the same elevations, the bacterial community was significantly different between the Apr.L and Oct.L groups (ANOSIM, *R* = 0.5021, *p* = 0.001), between the Apr.M and Oct.M groups (ANOSIM, *R* = 0.4904, *p* = 0.001), and between the Apr.H and Oct.H groups (ANOSIM, *R* = 0.4678, *p* = 0.001). During the flowering period, the bacterial community was significantly different between the Apr.L and Apr.M groups (ANOSIM, *R* = 0.214, *p* = 0.019) and between the Apr.L and Apr.H groups (ANOSIM, *R* = 0.3966, *p* = 0.001). However, no differences were observed between the Apr.M and Apr.H groups (ANOSIM, *R* = 0.01475, *p* = 0.332). Moreover, no significant differences were seen in the bacterial community between different elevations during the deciduous period. This indicates that the growing period had a larger effect on the rhizosphere bacterial community than elevation.

**Figure 6:**
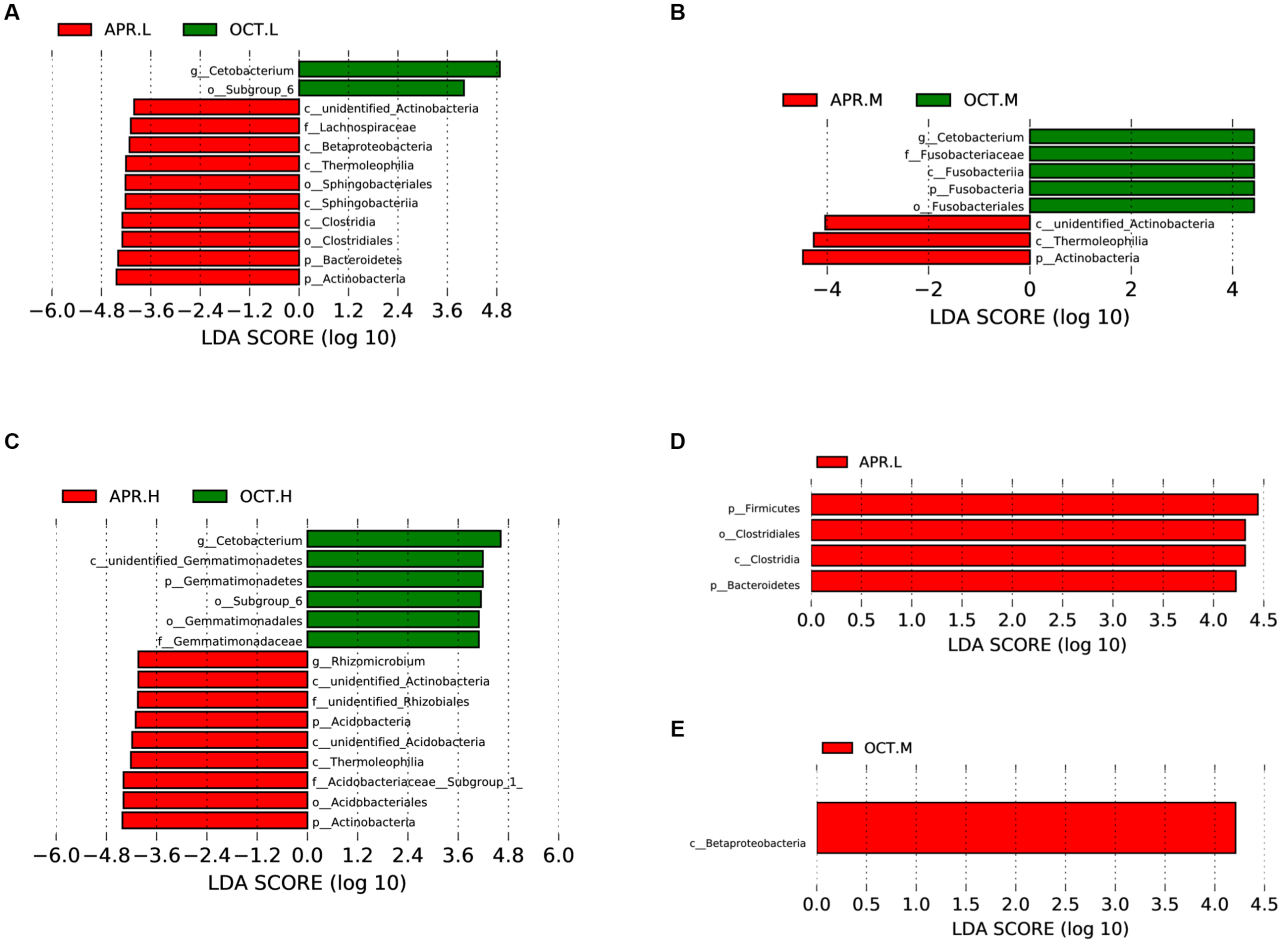
Differences in the composition of *R. mucronulatum* rhizosphere bacteria analyzed by LEfSe. LEfSe analysis (LDA > 4), differential bacterial communities of rhizosphere bacteria at low **(A)**, medium **(B)** and high **(C)** elevations in the flowering phase and the deciduous period; differential bacterial communities of rhizosphere bacteria at different elevations in the flowering period **(D)** and the deciduous period **(E)** (*n* = 9 per treatment). Apr.L, flowering period low elevation; Apr.M, flowering period medium elevation; Apr.H, flowering period high elevation; Oct.L, deciduous period low elevation; Oct.M, deciduous period medium elevation; Oct.H, deciduous period high elevation.

Using LEfSe analysis, we compared the differences in the richness of rhizosphere bacteria at the same elevation during the flowering and deciduous periods (Figures 6A–C). At the phylum level, Actinobacteria, Bacteroidetes, and Acidobacteria were significantly more abundant in flowering-period rhizosphere soil than in deciduous-period soils, and Fusobacteria and Gemmatimonadetes were significantly more abundant in deciduous-period rhizosphere soils than in flowering-period soil. At the family level, Lachnospiraceae and Acidobacteriaceae were significantly more abundant in the flowering period rhizosphere soil than in deciduous-period, and Fusobacteriaceae and Gemmatimonadaceae were significantly more abundant in deciduous-period rhizosphere soils than in flowering-period soil. At the genus level, *Rhizomicrobium* was significantly more abundant in flowering-period rhizosphere soil than in deciduous-period soil, and *Cetobacterium* was significantly more abundant in deciduous-period rhizosphere soil than in flowering-period soil. Additionally, we compared the differences in rhizosphere bacteria richness at different elevations in the flowering (Figure 6D) and deciduous periods (Figure 6E). Firmicutes (both Clostridia and Clostridiale belong to Firmicutes) and Bacteroidetes were significantly more abundant at low elevations than at other elevations in the flowering period. Betaproteobacteria was significantly more abundant at middle elevations than at other elevations in the deciduous period. Elevation had less of an effect on the difference in rhizosphere bacteria compared to the growing period.

### 3.5. Co-occurrence network analysis of *R. mucronulatum* rhizosphere bacteria

**Figure 7:**
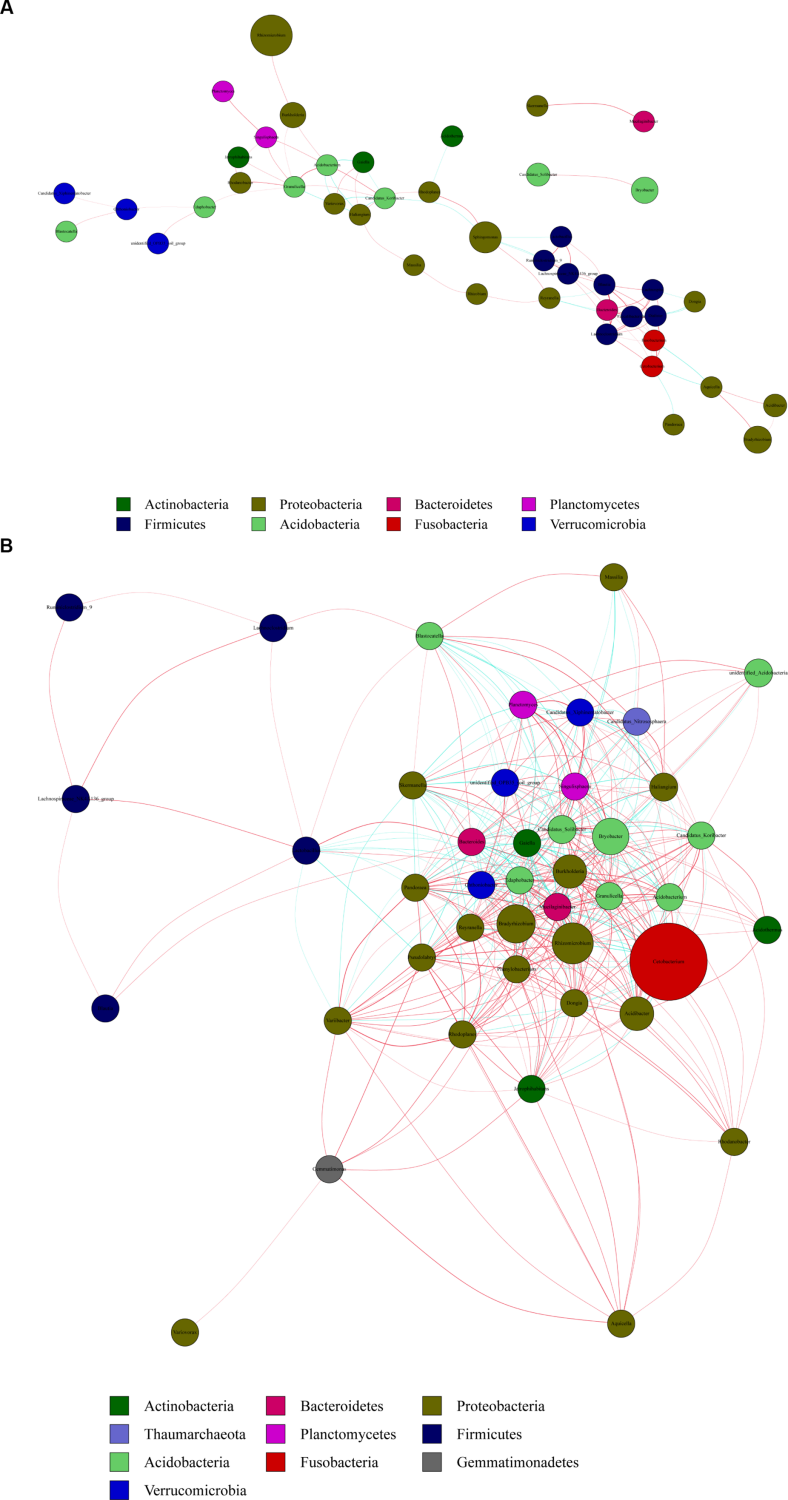
Network diagram of genus level correlations of rhizosphere bacteria at flowering **(A)** and deciduous **(B)** periods of *R. mucronulatum*. Red line represents positive correlation, green line represents negative correlation (*p* < 0.01). The nodes with the same color represent the same phylum, each node represents a genus, and the node size represents the average relative abundance of the genus.

We created co-occurrence network maps based on bacterial richness at the genus level in *R. mucronulatum* rhizosphere soil. The network for flowering period (Figure 7A) consisted of 43 nodes (genera) and 58 edges (significant correlation between genera), and the network for the deciduous period (Figure 7B) consisted of 43 nodes and 372 edges, indicating a stronger correlation between rhizosphere bacterial communities in deciduous period. The network at the phylum level during the flowering period, Proteobacteria were significantly correlated with six phyla and most closely associated with non-Proteobacteria bacterial communities. Verrucomicrobia was only significantly correlated with the Acidobacteria phylum. The network at the phylum level during the deciduous period, Proteobacteria were significantly correlated with nine phyla and most closely associated with non-Proteobacteria bacterial communities. The network at the genus level during the flowering period, there was a significant positive correlation between *Rhizomicrobium* and *Burkholderia*. The network at the genus level during deciduous period, both *Rhizomicrobium* (correlation with 30 genera significantly, *p* < 0.01) and *Cetobacterium* (correlation with 26 genera significantly, *p* < 0.01) were located in the densest area of the connecting line, with significant correlations between numerous genera. The density of the network diagram was 0.0635 in the flowering period and 0.342 in the deciduous period, and the association between genera was less close in the flowering period than in the deciduous period. Proteobacteria was the phylum most closely associated with other bacterial communities, in the flowering and deciduous periods, and *Rhizomicrobium* was the dominant genus in both periods.

### 3.6. Correlation analysis of *R. mucronulatum* rhizosphere bacteria with environmental factors

**Figure 8:**
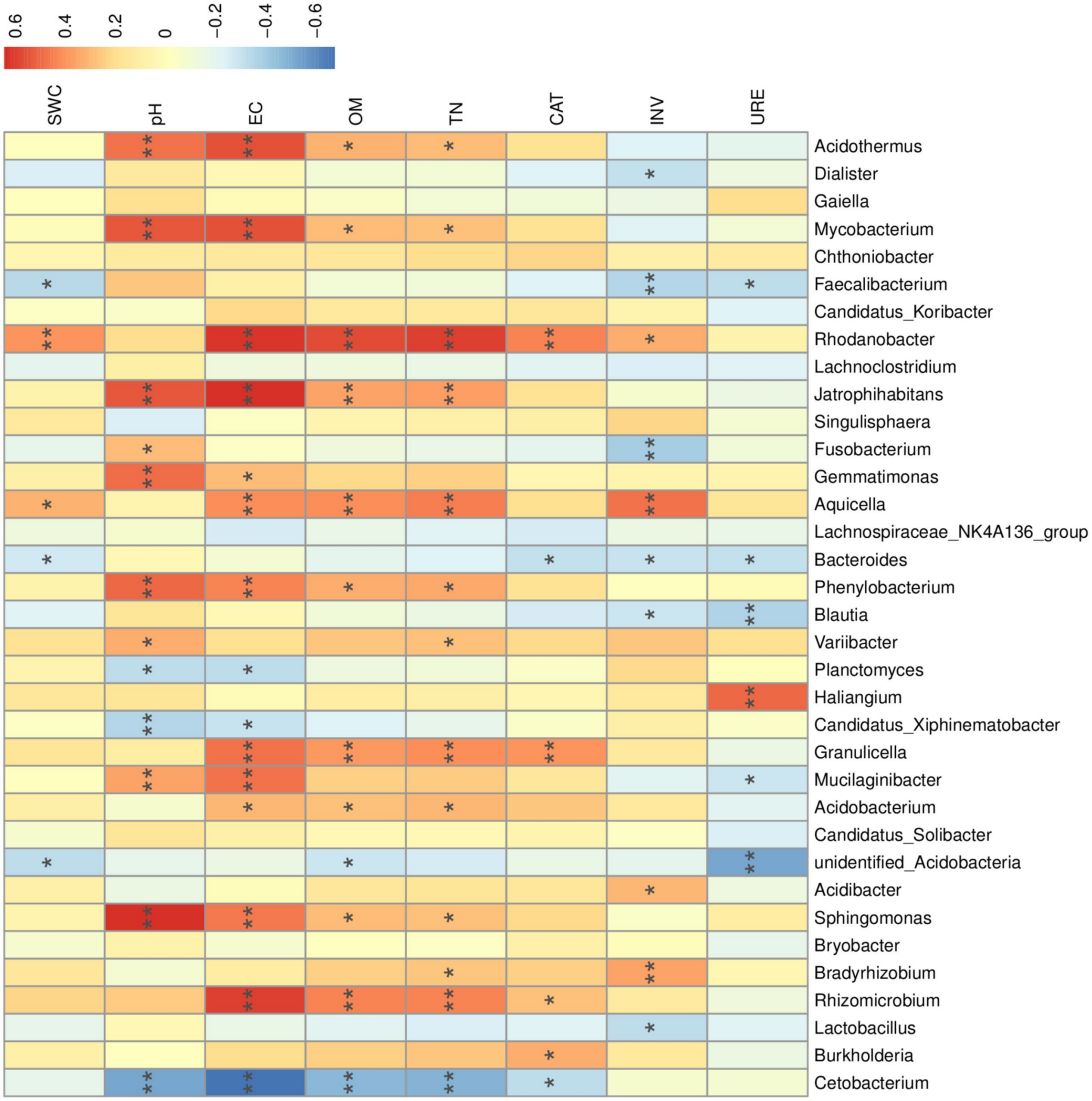
Correlation analysis between environmental factors and *R. mucronulatum* rhizosphere bacteriophage genera. The top 35 genera in terms of abundance were selected, with blue representing negative correlations and red representing positive correlations. Significant differences were labeled as * and ** for *p* < 0.05 and *p* < 0.01, respectively. EC, electrical conductivity; pH, soil pH; SWC, soil water content; OM, organic matter; TN, total nitrogen; CAT, soil catalase; INV, soil sucrose converting enzyme; URE, soil urease.

**Figure 9:**
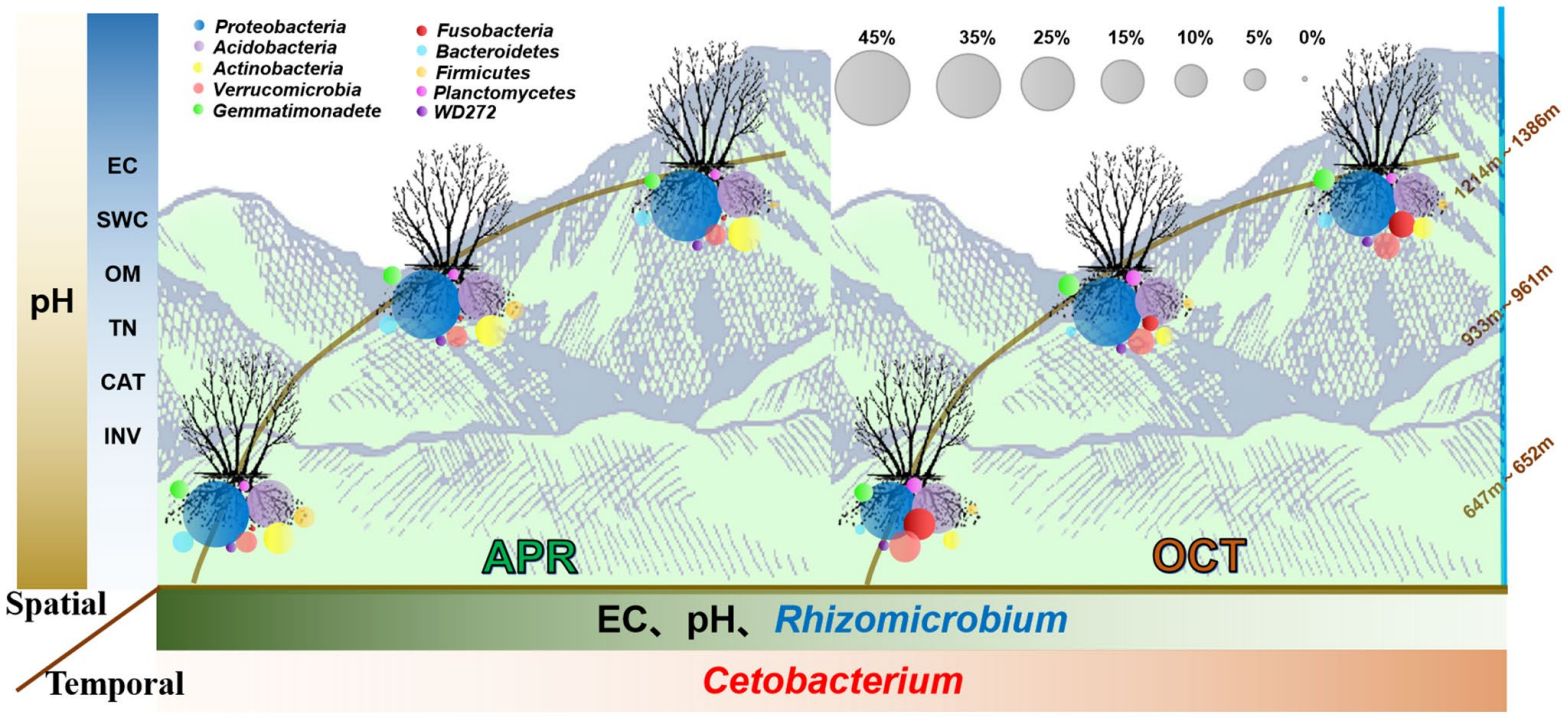
Trends of bacterial communities and soil characteristics in the rhizosphere of *R. mucronulatum* with growth period and elevation. The colour shades of the boxes to the left and below the image indicate trends in soil physicochemical property content, enzyme activity and relative abundance of bacterial with elevation or growth period. The darker the color, the higher the soil physicochemical property content, enzyme activity and relative abundance of bacteria. The circles of the same colour in the figure represent the same phylum and the size of the circles is proportional to their relative abundance.

We performed a nonparametric Spearman rank correlation analysis of environmental factors with the top 35 genera in terms of abundance (Figure 8). Soil pH and EC showed significant positive correlations with *Mycobacterium* spp., *Acidothermus spp.*, *Phenylobacterium spp.*, *Sphingomonas spp.*, *Mucilaginibacter spp.*, and *Jatrophihabitans spp.* The correlations between soil OM and TN and the high abundance of bacterial genera were generally consistent. OM and TN showed significant positive correlations with *Rhodanobacter* spp., *Rhizomicrobium* spp., *Jatrophihabitans* spp., *Aquicella* spp., and *Granulicella* spp. Many highly abundant genera within the dominant phyla Proteobacteria and Actinobacteria were significantly affected by soil pH, EC, OM, and TN. *Cetobacterium* spp. of the Fusobacteria phylum were significantly and negatively correlated with soil pH, EC, and OM. Soil pH, EC, OM, and TN may be the main factors affecting the *R. mucronulatum* rhizosphere bacterial community (Figure 9).

## 4. Discussion

### 4.1. Effects of a temporal and spatial variation on the rhizosphere soil properties of *R. mucronulatum*

The root absorbs nutrients from the soil to provide plants with the mineral nutrients and energy needed for growth (Wang et al., 2019; Gong et al., 2020). Soil is where the root grows and guarantees healthy plant growth. In our study, SWC increased with elevation during different periods, which is consistent with the findings of Bryant et al. (2008). At both the middle and high elevations, SWC was significantly higher in the deciduous period than in the flowering period, mainly owing to the decrease in temperature at high elevations, the increase in air humidity, and the decrease in evaporation of water from the soil. The temperature is lower in the deciduous period than in the flowering period and thus has an effect on SWC. The growth of *R. mucronulatum* has certain requirements for soil pH and benefits from acidic soil. The pH of rhizosphere soil was higher in the flowering period than in the deciduous period and decreased with increasing elevation. In contrast, the deciduous period had the highest soil pH at the middle elevation. There are two reasons for this result: (1) the differences in root exudation of *R. mucronulatum* in different growth periods and (2) the high precipitation at high elevations, which leads to leaching of alkaline cations. Sasse et al. (2018) found that root exudation varied with the growth period in Arabidopsis, rice, and oats; Yuan et al. (2014) found that soil pH decreased with elevation in highland areas with higher precipitation. Both findings are generally consistent with ours. Soil OM was mostly acidic, and the rhizosphere soil OM content in different growth periods of *R. mucronulatum* increased with elevation, which was one of the reasons for the decrease in soil pH. The variation pattern of soil OM with elevation is consistent with the findings of others (Han et al., 2010; Siles et al., 2017; Zhang et al., 2021). Soil OM and TN are important nutrient sources for *R. mucronulatum*. Soil EC can reflect the actual conditions of soil salinity under certain conditions and can affect the conversion of soil nutrients. The growth period had less effect on EC, OM and TN in rhizosphere soils than elevation. Soil OM in forests is primarily derived from plants and microbial residues. Plants have a significant positive effect on soil properties and can improve soil quality (Gong et al., 2015). At high elevations, a large amount of apoplankton exists, which provides OM to the soil. Additionally, the activities of various enzymes that use this OM as a substrate gradually increase, with CAT, INV, and URE all showing maximum activity at high elevations.

We also found significant correlations between SWC, EC, OM, TN, and enzymatic activities of *R. mucronulatum* rhizosphere soil in the flowering and deciduous periods. Enzymes in soil play an important role in OM formation and degradation, exogenous material decomposition, and nutrient cycling (Song et al., 2012; Xu et al., 2015; Soares and Rousk, 2019; Wang et al., 2022b). SWC acts as a solvent or participates directly in chemical reactions. At the same time, enzymes in the soil influence the process of carbon, nitrogen, and phosphorus cycling, as well as nutrient decomposition and transformation in soil ecosystems (Brockett et al., 2012). The relationship between soil pH and other soil parameters was negative or not significantly correlated, and it was only significantly positively correlated with URE in the flowering period. This may be because pH affects the ionization and deionization of the acidic group in the active center of the enzyme protein, and because URE exhibits more activity when the enzyme-active center is in a suitable ionized state.

### 4.2. Effects of a temporal and spatial variation on the composition and diversity of *R. mucronulatum* rhizosphere bacterial communities

Soil microbial communities account for one-quarter of the Earth’s total biodiversity and are the most biodiverse communities in the biosphere (Guerra et al., 2021; Sokol et al., 2022). These soil microorganisms play a vital role in the decomposition and transformation of OM and in nutrient cycling (Hirsch and Mauchline, 2015; Tian et al., 2021; Wang et al., 2021). Because of the interaction between various environmental factors and the influence of key environmental factors on soil microorganisms, the soil microbial community structure shows a pattern of temporal and spatial changes. We used 16S rRNA sequencing technology to analyze the characteristics of the *R. mucronulatum* rhizosphere bacterial community. Ortiz et al. (2020) found that the dominant phyla in the rhizosphere soil of *Tabebuia heterophylla* were Proteobacteria, Actinobacteria, and Verrucomicrobia. Vargas et al. (2023) found that the dominant phyla in the rhizosphere soil were all Proteobacteria, Acidobacteria, and Actinobacteria at different soil fertility levels. We found that changes in temporal and spatial sampling did not induce changes in the dominant phyla in *R. mucronulatum* rhizosphere soil, i.e., Proteobacteria, Acidobacteria, Actinobacteria, and Verrucomicrobia (relative abundance >5%). This generally agrees with the results of Ortiz et al. and Vargas et al. Many studies have confirmed the ability of bacteria from the Proteobacteria, Acidobacteria, and Actinobacteria phyla to promote primary root elongation, increase root branching, inhibit pathogens, and degrade lignin to promote plant growth (Lugtenberg and Kamilova, 2009; Tan et al., 2022; Zuo et al., 2022b).

Further analysis revealed significant differences in the Shannon diversity index and Chao1 richness index of the bacterial community during the flowering and deciduous periods of *R. mucronulatum*. Zhao et al. (2022) demonstrated that the growth period could significantly change the bacterial community structure. Tang et al. (2020) suggested that elevation is the dominant factor driving bacterial community differentiation and that rhizosphere bacterial communities change significantly with the elevation gradient. In our study, the effect of growth periods on the diversity of the rhizosphere bacterial community was greater than the effect of the elevation gradient on the diversity of the rhizosphere bacterial community. Guo et al. (2020) studied temperate montane forests in China and found that rhizosphere microbial changes along the elevational gradients were caused more by plant characteristics. Plant root exudation is the main source of carbon and nitrogen for rhizosphere microbial life activities. It determines the species and number of rhizosphere microbes, and it varies at different periods of plant growth and development, causing changes in the structure of the rhizosphere microbial community (Schreiter et al., 2014). Plant litter can also directly or indirectly influence the composition of rhizosphere microorganisms (Schlatter et al., 2015; Guo et al., 2020). Temperature changes can affect root exudation directly by affecting plant root growth and physiological activity and can affect it indirectly by regulating soil water and nutrient effectiveness (Yin et al., 2013; Kaiser et al., 2015; Zhang et al., 2016). At the onset of the warm season, plants at lower elevations recover first, accelerating photosynthetic rates and producing secretions from roots that affect the rhizosphere microbial community. When the temperature decreases in the deciduous period, plant roots become less able to absorb nutrients, so photosynthesis and respiration are weakened. Root exudation also changes significantly from the warm season. The physiological metabolism of the root of *R. mucronulatum* was significantly altered in different growth periods. The Shannon diversity index of the bacterial community was significantly higher in the flowering period than in the deciduous period, possibly owing to the decrease in root exudation, which depends on some of the flora in the deciduous period. This led to a decrease in the relative abundance of these floras and thus caused changes in the structure of the rhizosphere bacterial community.

When comparing the rhizosphere bacterial diversity of *R. mucronulatum* at the same elevation in the flowering and deciduous periods, we observed that more bacterium of dominant phyla were enriched in the flowering rhizosphere soil samples than in samples from the deciduous period. Actinobacteria was significantly more abundant in the flowering-period rhizosphere soil samples at different elevations than in the deciduous-period rhizosphere soil samples. Studies have revealed that Actinobacteria provide plants with the nutrients required to promote plant growth, respond to biotic stress, and defend against plant pathogens (Manigundan et al., 2022). The number of phyla that differed significantly among elevation groups in the flowering or deciduous period was low. In the genus-level correlation network diagram, the deciduous-period rhizosphere bacterial communities were more closely linked to each other, possibly because of changes in root exudation. *Cetobacterium* and *Rhizomicrobium*, the dominant genera of *R. mucronulatum* rhizosphere bacteria, reached relative abundances of 0.079 and 0.023, respectively, in the deciduous period, and they were significantly negatively correlated. The relative abundance of *Rhizomicrobium* spp. in the flowering period was 0.038, which was higher than that in the deciduous period. When changes occur in root exudation, the relative abundance of microorganisms benefiting from chemical changes near the roots increases, and their metabolic activity also increases, and vice versa (Schreiter et al., 2014). The temperature decreases in October, leading to a decrease in the metabolic activity of *R. mucronulatum*. *Rhizomicrobium* may be sensitive to changes in plant root exudation, and changes in root exudation and increases in the relative abundance of *Cetobacterium* in the deciduous period lead to a decrease in the relative abundance of *Rhizomicrobium*.

### 4.3. Relationship between the integrated physicochemical properties of rhizosphere soil and bacterial communities

Many soil microorganisms are attached to the narrow space of the rhizosphere, and they are frequently active in the rhizosphere area, transforming organic substrates, releasing minerals, and changing soil properties, affecting plant growth (Zhao et al., 2015). Rhizosphere bacteria can have a significant influence on plant health, nutrient uptake, and soil properties (Dundas and Dinneny, 2022). Enzymes in the soil can directly affect soil physicochemical properties and indirectly influence rhizosphere bacterial communities by decomposing OM. In this study, soil pH, EC, OM, and TN significantly affected the composition of the rhizosphere bacterial community, and enzyme activity in soil had a relatively less effect on the rhizosphere bacterial community. Soil pH has a wide range of influences on rhizosphere bacterial communities, involves many phyla, and is one of the main factors affecting the structure of soil microbial communities. EC, OM, and TN in soil were significantly and positively correlated with *Rhodanobacter*, *Rhizomicrobium*, and *Sphingomonas* of Proteobacteria and *Mycobacterium* of Actinobacteria. Numerous genera within the Proteobacteria and Actinobacteria phyla have been demonstrated to promote plant growth directly or indirectly (Yang et al., 2017; Kalntremtziou et al., 2023; Panchenko et al., 2023). Soil OM is the primary source of soil microbial carbon and nitrogen (Floch et al., 2009). The quality and availability of OM in soil largely determine the survival ability of soil microorganisms (Nguyen and Marschner, 2015; Bhattacharyya et al., 2022). *Rhizomicrobium* is a nitrogen-fixing bacterium and is involved in phosphate and phosphite metabolism, affecting plant growth (Li et al., 2021; Xun et al., 2021). The high enrichment of *Rhizomicrobium* spp. in the flowering period can play a positive role in the nutritional growth of *R. mucronulatum*. Moreover, *R. mucronulatum* rhizosphere soil characteristics have a strong influence on the structure of rhizosphere bacterial communities.

## 5. Conclusion

We investigated the effects of temporal and spatial variation on the rhizosphere bacterial diversity of *R. mucronulatum* by analyzing the rhizosphere soil of *R. mucronulatum* in Beijing Yunmeng mountain National Forest Park, Beijing. It was found that the diversity of the rhizosphere bacterial community of *R. mucronulatum* changed significantly with the change of growing period. The effect of soil physicochemical properties on the rhizosphere bacterial community was higher than the enzyme activity in the soil. Bacteria of the genus *Rhizomicrobium* were able to positively promote the nutritional growth of *R. mucronulatum*. A comprehensive analysis of the effect of temporal and spatial variation on the rhizosphere soil properties and rhizosphere bacterial diversity of *R. mucronulatum* was conducted to lay the foundation for an in-depth understanding of the changing patterns of the rhizosphere microecological environment of *R. mucronulatum*.

## Data availability statement

The datasets presented in this study can be found in online repositories. The names of the repository/repositories and accession number(s) can be found at: https://www.ncbi.nlm.nih.gov/, PRJNA950208.

## Author contributions

JC conceived and designed the experiments and performed the sample collection. TZ collected some samples. SW analyzed the data and wrote the paper. HZ and KZ analyzed the data too. All authors contributed to the article and approved the submitted version.

## Funding

This study was funded by the Beijing Innovation Consortium of Agriculture Research System (project number: BAIC09-2022).

## Conflict of interest

The authors declare that the research was conducted in the absence of any commercial or financial relationships that could be construed as a potential conflict of interest.

## Publisher’s note

All claims expressed in this article are solely those of the authors and do not necessarily represent those of their affiliated organizations, or those of the publisher, the editors and the reviewers. Any product that may be evaluated in this article, or claim that may be made by its manufacturer, is not guaranteed or endorsed by the publisher.
